# Morphological, thermal and mechanical properties of recycled HDPE foams via rotational molding

**DOI:** 10.1177/0021955X211013793

**Published:** 2021-04-29

**Authors:** Yao Dou, Denis Rodrigue

**Affiliations:** Department of Chemical Engineering and CERMA, Université Laval, Quebec City, QC, Canada

**Keywords:** High density polyethylene, rotomolding, recycling, chemical blowing agent, properties

## Abstract

In this study, foamed recycled high density polyethylene (rHDPE) parts were produced by rotational molding using different concentration (0 to 1% wt.) of a chemical blowing agent (CBA) based on azodicarbonamide. From the samples produced, a complete morphological, thermal and mechanical characterization was performed. The morphological analysis showed a gradual increase in the average cell size, while the cell density firstly increased and then decreased with increasing CBA content. As expected, increasing the CBA content decreased the foam density as well as the thermal conductivity. Although increasing the CBA content decreased both tensile and flexural properties, the impact strength showed a similar trend as the cell density with an optimum CBA content around 0.1% wt. Finally, neat rHDPE samples were also produced by compression molding. The results showed negligible differences between the rotomolded and compression molded properties indicating that optimal rotomolding conditions were selected. These results confirm the possibility of using 100% recycled polymers to produce rotomolded foam parts.

## Introduction

In the last decades, rotational molding (rotomolding) received a great deal of interest due to its simple processing tools, low machinery cost and limited waste generation.^1–4^ The main reason is the technology allowing to easily produce large one-piece hollow and seamless products such as industrial storage tanks, automotive parts, furniture and several other items. Compared with other plastic processing techniques, like injection and blow molding, there is no pressure involved in rotomolding meaning that the molds can be very thin and generally inexpensive. Furthermore, rotomolding can more easily handle complex shaped articles with uniform wall thicknesses. These features make rotomolding one of the fastest-growing polymer processes in the plastic industries over the last few years.^5–7^

Over the last decades, scientific and industrial research has been increasingly focused on polymeric foams since their cellular structure offers unique physical properties while reducing the weight (amount of materials consumed). Foams have improved insulation properties, cushioning properties and outstanding stiffness-to-weight ratios contributing to several applications, such as thermal insulation, buoyancy, packaging and gaskets.^8–10^ In rotational molding, foams with skin-core morphologies can be used to manufacture creative and high-value articles without specialized equipment. The hollow structure of rotomolded products can also be used to overcome some limitations related to low mechanical and shock mitigation properties.^11–13^

Still today, about 90% of all parts produced by rotational molding are based on different grades of polyethylene including low density polyethylene (LDPE), linear low density polyethylene (LLDPE), high density polyethylene (HDPE) and cross-linked low density polyethylene (XLDPE), because they have low melting temperature, low cost and high temperature resistance.^14–17^ Furthermore, several studies have been conducted on the foaming mechanisms of polyethylene.^18–21^ As reported in the literature, there is some agreement among researchers regarding the stages of a typical foaming process: cell nucleation, cell growth (cell coalescence and cell coarsening) and cell stabilization. But very few investigations focused on the properties of polyethylene foams produced by rotomolding.^22–25^ For example, Archer et al. reported a linear decrease in both flexural modulus and compressive strength with decreasing metallocene catalyzed LLDPE foam density.^23^ In our previous work, we reported that increasing the chemical blowing agent (CBA) content led not only to lower tensile and flexural moduli, but also to lower tensile strength and elongation at break, which can be associated with lower density and larger cell size.^25^

With the ever-increasing consumption of polyethylene products in recent decades, a large number of solid wastes are generated causing serious environmental issues worldwide since they do not easily degrade and remain in the environment for a long time.^26,27^ Hence, seeking new ways to reuse recycled polyethylene is essential to minimize the amount of waste. HDPE is a typical example of available recycled polyethylene which can have several potential applications because of its good dimensional, mechanical and thermal stability.^27,28^ Moreover, the average cost of producing plastic products from recycled HDPE (rHDPE) is approximately 31–34% lower than that from virgin HDPE.^29^ Consequently, using rHDPE not only decreases waste disposal issues, but also reduces the cost of HDPE based products.

Dvorak studied the possibility of using recycled HDPE instead of virgin HDPE in rotational molding.^30^ She found that rHDPE initially produced by rotomolding and injection molding had suitable melt flow index to be reused in rotomolding. Chaisrichawla and Dangtungee blended different ratios of virgin LLDPE and recycled HDPE from blowing processes to manufacture rotomolded products (septic tanks).^31^ Nevertheless. To the best of our knowledge, no study was performed/published on recycled HDPE foams produced by rotomolding. Consequently, the main objective of this work is to produce foamed and unfoamed rotomolded parts based on recycled high density polyethylene. In particular, the effect of chemical blowing agent content is investigated to determine its relation with foam density and cellular structure (cell size and cell density), and then to further determine its effect on the thermal (conductivity) and mechanical properties (tensile, flexural and impact) of rHDPE foams. Finally, to determine if the optimal rotomolding conditions were selected, neat solid rHDPE samples are also produced by compression molding to compare the properties of samples from both processing methods.

## Experimental

### Materials

**Figure 1.**
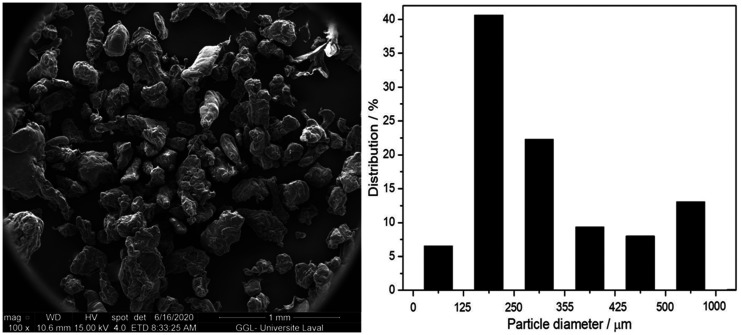
Typical scanning electron microscopy image of the rHDPE powder used (left) with its particle size distribution (right).

The recycled HDPE used was provided by Service de Consultation Sinclair (Drummondville, QC, Canada). This material was supplied in flakes coming from recycled solid HDPE bottles. The material was then pulverized using a model PKA18 pulverizer (Powder King, Phoenix, AZ, USA) The powder was then characterized to get its melt flow index (6.7 g/10 min at 2.16 kg/190 °C) and its peak melting temperature (123 °C) as determined via differential scanning calorimetry (DSC at 10 °C/min). The final powder morphology is presented in Figure 1. For foaming, an exothermic chemical blowing agent (CBA) based on activated azodicarbonamide was used: Celogen 754 A (powder) from Chempoint (USA). Its peak decomposition temperature is 164 °C as determined via DSC.

### Rotational molding

**Figure 2.**
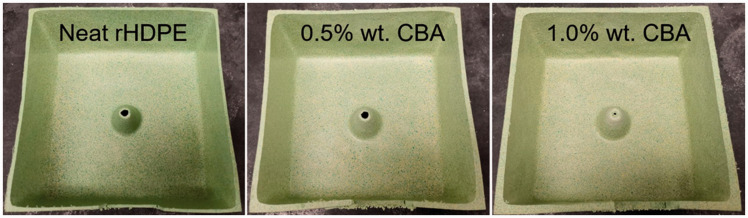
Typical examples of the rotomolded rHDPE parts (cut samples).

A series of rHDPE foams were prepared by using different CBA contents (0.1, 0.2, 0.3, 0.4, 0.5, 0.6, 0.7, 0.8, 0.9 and 1% wt.) to compare with the unfoamed matrix (0% wt.). As the CBA must be thoroughly dispersed in the rHDPE powder prior to charging the mold, all the materials were dry-blended in a high-speed mixer LAR-15LMB (Skyfood, USA) at 3320 rpm with fixed intervals of 1 min mixing time and 1 min cooling time repeated 5 times. For processing, a laboratory-scale biaxial rotational molding machine was used (MedKeff-Nye Roto-Lab model 22, Barberton, OH, USA). Rotationally molded parts were manufactured with a cubic aluminum mold of 3.6 mm wall thickness and an internal side length of 19 cm. Before loading the material, a demolding agent (Trasys 420, DuPont, Midland, MI, USA) was applied to the internal mold surface. A circular vent (diameter = 10 mm) was filled with glass wool to prevent powder losses. After several preliminary runs, the optimum processing conditions were: a 3:4 speed ratio (major axis:minor axis), a heating time of 18 min with an oven (electrically heated) temperature of 270 °C and a cooling time of 30 min with forced air (blowing fans). Finally, the mold was opened and the part was demolded. To perform the characterizations, samples were directly cut in the molded parts (Figure 2). All the samples were produced using 660 g, so the final part thickness (2.4 to 6.2 mm) depends on the CBA content (final density).

### Compression molding

To produce the compression molded rHDPE parts, 35 g of the powder was placed in a mold with dimensions of 110 × 110 × 3 mm^3^. The material was compression molded in an automatic Carver hydraulic press model Autoseries 3893 (Carver Inc., USA) at 190 °C with a constant force of 2200 kg for 10 min, and finally the mold was cooled by water circulation to 60 °C before removing the pressure and demolding.

### Differential scanning calorimetry

Differential scanning calorimetry (DSC) studies were performed on a DSC-7 from Perkin-Elmer (USA) equipped with a thermal analysis controller TAC7/DX. About 15 mg of rHDPE powder or CBA were weighed and placed in a sealed aluminum pan. The measurements were carried out with a scanning rate of 10 °C/min between 50 and 200 °C under a flow of dry nitrogen (20 mL/min). The first heating cycle of rHDPE powder was only used to delete its thermal history and was not analyzed.

### Thermogravimetric analysis

Thermogravimetric analysis (TGA) was carried out on a Q5000IR TGA analyzer (TA Instruments, USA). The scans were performed from 50 to 800 °C at a rate of 10 °C/min with a gas (nitrogen) flow rate of 25 mL/min.

### Morphological characterization

The foamed rHDPE parts were cryogenically fractured (liquid nitrogen) and micrographs of the exposed cross-sections were taken using a scanning electron microscope (SEM) (FEI Inspect F50, USA). Foam morphology characterization was investigated based on two parameters: cell size (*D*) and cell density ( $N_{f}$ ). The average cell size with standard deviation was measured by the ImageJ software (US National Institutes of Health, USA). Cell density ( $N_{f}$ ), which is defined as the number of cells per cubic centimeter of foam, was calculated according to the method of Kumar and Weller as:^32^

(1) $$N_{f}={\left.\left(\frac{n}{A}\right.\right)}^{3/2}$$

where *n* is the number of cells in a micrograph and *A* is the area of the micrograph in cm^2^.

### Density and hardness

To determine the density, each sample was cut into cubes of different dimensions (measured with a caliper having a resolution of ± 0.01 mm), weighed (MX-50 moisture analyzer, A&D, Tokyo, Japan) and determined using a gas (nitrogen) pycnometer Ultrapyc 1200e (Quantachrome Instruments, USA) to compare. Hardness (Shore A and Shore D) was obtained by a PTC Instruments (USA) Model 306 L and Model 307 L (ASTM D2240), respectively. The results reported are the average and standard deviation of a minimum of 5 samples.

### Thermal conductivity

The effective thermal conductivity (*k*) of the samples was determined by an in-house built thermal conductivity analyzer following ASTM E1225. The rotomolded parts were cut into square samples (50 × 50 mm^2^) and their thickness (*d* ± 0.01 mm) was measured using a digital caliper (Mastercraft, Canada). The samples were sandwiched between thin aluminum foil sheets to limit the surface thermal resistance during measurement by fixing the hot (top = *T_h_*) and cold (bottom = *T_c_*) plate at 33 and 13 °C respectively (20 °C of temperature difference giving an average of 23 °C = room temperature) using water cooled Pelletier plates (Model K20, Haake, Germany). These temperatures were measured using thermistances (TC-720, TE-Technology, USA) and the heat flux (*Q*) was determined by a PHFS-01 heat flux sensor (Flux Teq LLC, USA). Each sample was tested three times to measure the average thermal conductivity with their respective standard deviations. For each experiment, equilibrium values were obtained after about 30 min. The thermal conductivity was determined as:

(2) $$k=\frac{\mathrm{QXL}}{\Delta T}$$

where *Q* is the heat flux (BTU/ft^2.^h), *X* is the conversion factor (BTU/ft^2.^h converts to W/m^2^ by multiplying by 3.1546), *L* is thickness of the specimen (mm) and *ΔT* is the temperature difference (20 °C).

### Mechanical properties

All the specimens were cut from the rotomolded parts and measured at room temperature. The tensile properties were conducted on dog bone samples according to ASTM D638 (type V) on an Instron (USA) model 5565 universal testing machine with a 500 N load cell. The crosshead speed was set at 10 mm/min and the values for tensile modulus, tensile strength and elongation at break are based on the average (± one standard deviation) of at least six samples.

Flexural tests (three-point bending) were performed according to ASTM D790 using a crosshead speed of 2 mm/min on an Instron (USA) universal tester model 5565 with a 50 N load cell. The span length was fixed at 60 mm. At least five rectangular samples (60 × 12.7 mm^2^) were used to report the average and standard deviation for the modulus.

Charpy impact strength was determined by a Tinius Olsen (USA) testing machine model Impact 104. At least ten rectangular specimens (60 × 12.7 mm^2^) were prepared according to ASTM D6110. The samples were notched (“V” shaped) by an automatic sample notcher model ASN 120 m (Dynisco, USA) at least 24 h before testing.

## Results and discussion

### Differential scanning calorimetry

**Figure 3.**
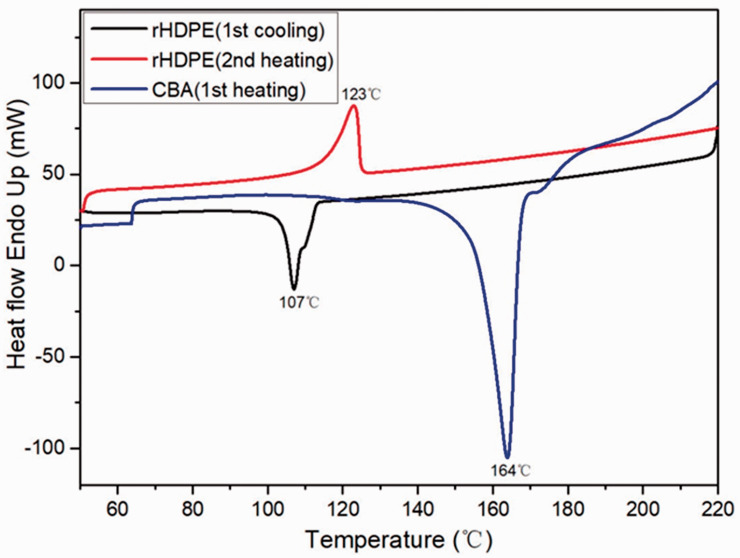
DSC thermograms of the rHDPE powder and CBA used.

Figure 3 presents the DSC thermographs of the rHDPE powder and CBA. The second heating cycle of rHDPE powder presents a single endothermic peak which confirms that the rHDPE is mainly alone in the resin. The peak melting temperature and crystallization peak temperature for rHDPE are 123 °C and 107 °C, respectively. Figure 3 also shows that the onset decomposition temperature of CBA is about 140 °C, while its peak decomposition temperature is 164 °C. Thus, the oven temperature in the heating cycle of rotomolding was set as 270 °C to ensure complete rHDPE melt and CBA decomposition.

### Thermogravimetric analysis

**Figure 4.**
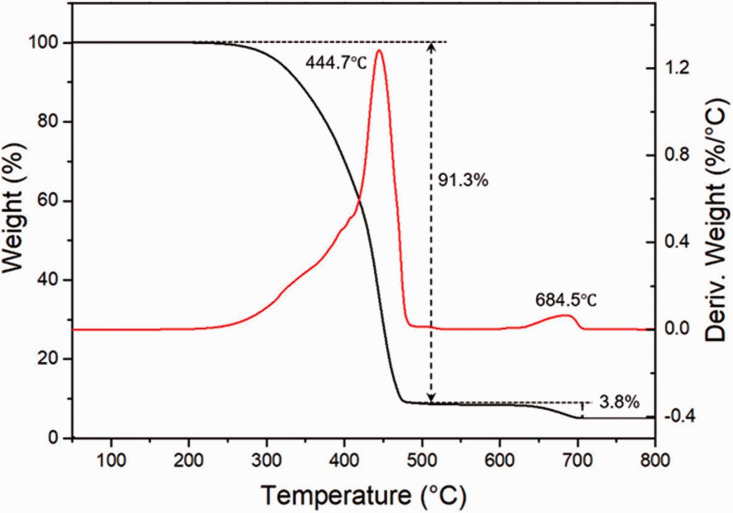
Typical TGA (black) and DTG (red) thermograms of the rHDPE powder used.

The TGA and derivative of thermogravimetry (DTG) curves obtained for the rHDPE powder under a nitrogen atmosphere are depicted in Figure 4. The rHDPE sample remains stable from 50 to 260 °C as no weight loss occurs. Above 260 °C, the decomposition starts until the sample is completely decomposed at 490 °C with a peak temperature at 445 °C. A weight loss of 91.3% was recorded in this zone which represents the thermal degradation of rHDPE. Then, around 3.8% weight loss is observed between 628 °C and 700 °C due to the decomposition of organic fillers. However, there is about 4.9% weight of residues which can be related to the presence of inorganic components in the rHDPE powder (different additives related to the post-consumer origin of the polymer).

### Morphological characterization

**Figure 5.**
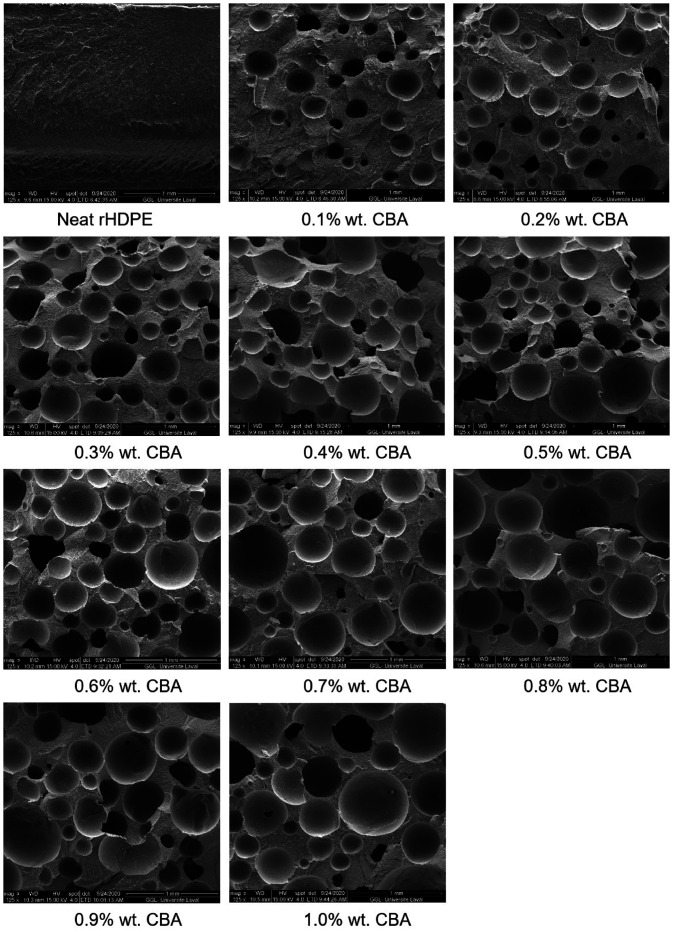
Typical morphologies of the rotomolded rHDPE parts with different CBA contents.

SEM images for the foams with different CBA contents at a low magnification (125x) are shown in Figure 5. Based on these images and their quantitative analysis, the average cell size and cell density are summarized in Table 1. As expected, the average cell size increases with increasing CBA content since more gas is available to blow the nucleated cells. For example, the average cell size increases from 0.191 to 0.349 mm when the CBA content increases from 0.1% to 1.0% wt. On the other hand, the cell density firstly increases at low CBA content (0.1 to 0.3% wt.), but then decreases at higher CBA content (0.3 to 1% wt.). This trend, leading to a maximum *N_f_* (optimum CBA content), represents a balance between the amount of gas generated and the thinner cell walls/higher internal cell pressure leading to cell coarsening and coalescence.^33^ There is also higher probability of gas loss with increasing CBA content.^34^ This observation is similar with our previous work where the cell density of LLDPE foams firstly increased at low CBA content (0.1 to 0.2% wt.), and then decreased with further CBA content increase from 0.2 to 1% wt.^25^

**Table 1.** Average cell size and cell density of rHDPE foams.

CBA content (% wt.)	Average cell diameter (μm)	Cell density (10^3^/cm^3^)
0.1	191 ± 81	14.3
0.2	192 ± 98	24.9
0.3	218 ± 96	28.5
0.4	241 ± 114	26.7
0.5	242 ± 129	24.0
0.6	272 ± 114	24.9
0.7	315 ± 165	21.3
0.8	328 ± 167	18.8
0.9	341 ± 154	15.8
1.0	349 ± 150	15.1

### Density and hardness

**Figure 6.**
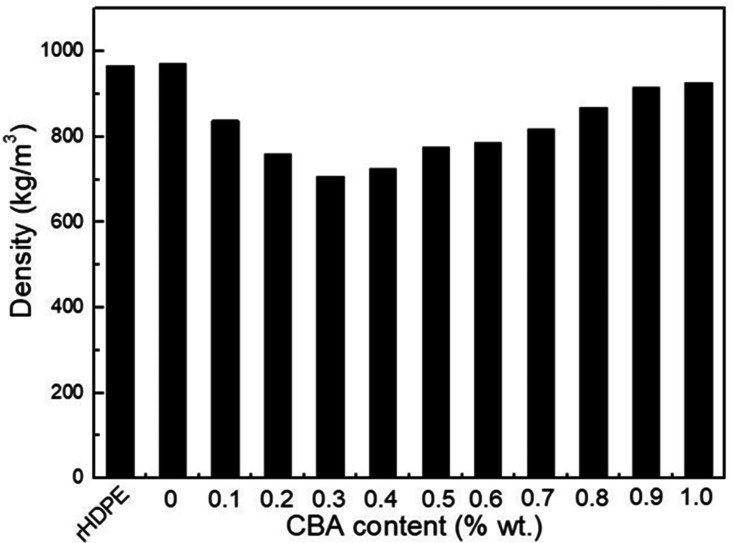
Density of rHDPE powder and rHDPE foams as a function of CBA content.

Figure 6 presents the density of the rHDPE powder and rHDPE foams with different CBA contents. Increasing the CBA content up to 0.3% wt. decreases (27%) the density from 0.976 to 0.707 g/cm^3^. The higher than expected density value (0.93 to 0.96 g/cm^3^) for the rHDPE might be coming from the recycled nature of the materials which may contain several additives and/or contamination to give its final color as shown in Figure 2 and the residues in Figure 4.^35,36^ However, the foam density increases from 0.707 to 0.914 g/cm^3^ between 0.3 and 1% wt. CBA. This increasing trend is attributed to cell instability (coalesce) and gas loss with increasing CBA content above the optimum value. Some of these defects (broken cell walls) can be seen in Figure 5.

**Figure 7.**
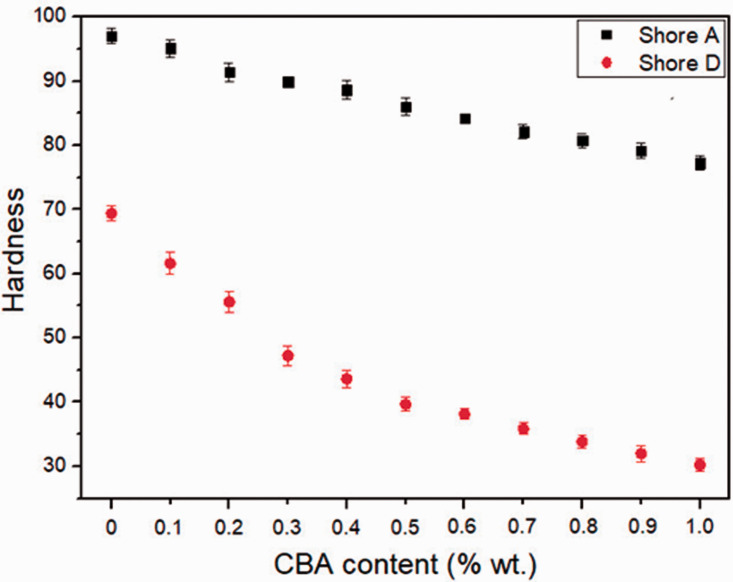
Hardness (Shore A and Shore D) of rHDPE foams as a function of CBA content.

Figure 7 shows that hardness (Shore A and Shore D) continuously decreases with increasing CBA content from 0% to 1.0% wt. In this case, the Shore A decreased from 97 to 77 (20 points difference), while the Shore D decreased from 69 to 30 (39 points difference). These trends are expected due to decreasing cell wall thickness (increasing cell size in Table 1) inside the foams, and the “soft” nature of gas cells occupying more space compared to the neat matrix (rHDPE).

### Thermal conductivity

Table 2 reports the effect of CBA content on the thermal conductivity. Both the thermal conductivities of neat rHDPE produced by compression molding and rotomolding are 243 mW/m^.^K which is lower than reported values of recycled HDPE (300 mW/m^.^K) in the literature.^37^ This might be associated with the rHDPE powder having some non-conductive (inorganic) components as observed via TGA (Figure 4), leading to lower thermal conductivity as it was the case for density (Figure 6). Nevertheless, increasing the CBA content led to lower thermal conductivity with the lowest value (165 mW/m^.^K) achieved at 1% wt. CBA. This trend is similar as hardness (Figure 7) and inversed to the average cell size (Table 1) indicating that cell size is the most important parameter here (gas contribution compared to the polymer contribution).

**Table 2.** Thickness and thermal conductivity (*k*) of rHDPE foams.

CBA content (% wt.)	Thickness (mm)	*k* (mW/m^.^K)
0^c^	2.31	243 ± 11
0^r^	2.35	243 ± 12
0.1	2.92	203 ± 18
0.2	3.19	201 ± 12
0.3	3.77	193 ± 4
0.4	4.56	191 ± 5
0.5	4.92	188 ± 4
0.6	5.00	187 ± 14
0.7	5.20	184 ± 6
0.8	5.53	175 ± 9
0.9	5.87	173 ± 7
1.0	6.13	165 ± 10

c: compression molded; r: rotational molded.

### Flexural properties

**Figure 8.**
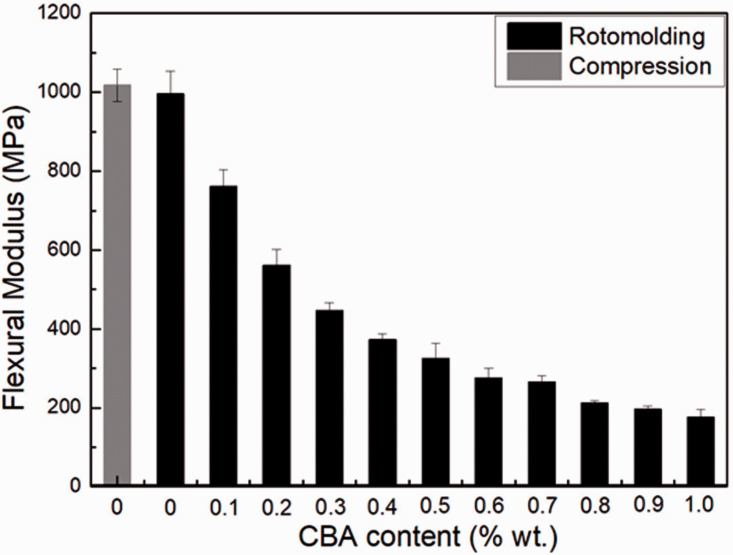
Flexural modulus of rHDPE foams as a function of CBA content.

Figure 8 presents the flexural modulus of the neat rHDPE parts and the foams. For both neat rHDPE samples, the flexural modulus of the compression molded sample is 1017 ± 41 MPa which is similar to the rotomolded one (996 ± 58 MPa) within experimental uncertainty. It can also be seen that the values substantially decrease with increasing CBA content. For example, the flexural modulus of the sample with 1.0% wt. CBA is 176 MPa, which represents a 82% decrease. Lower values for foams are related to less material being available (decreasing density in Figure 6) to sustain the applied stress and higher amount of cells collapse/larger cells (Table 1 and Figure 5).^38^

### Tensile properties

**Figure 9.**
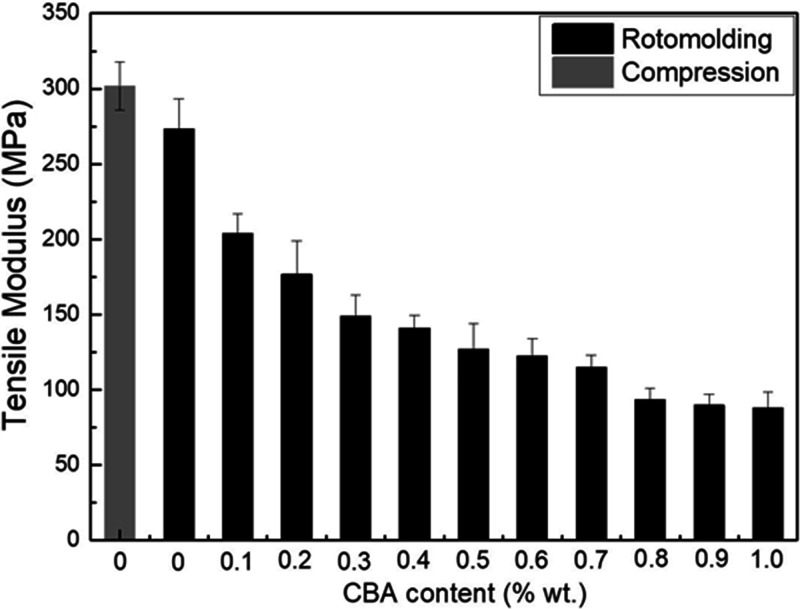
Tensile modulus of rHDPE foams as a function of CBA content.

**Figure 10. fig10-0021955X211013793:**
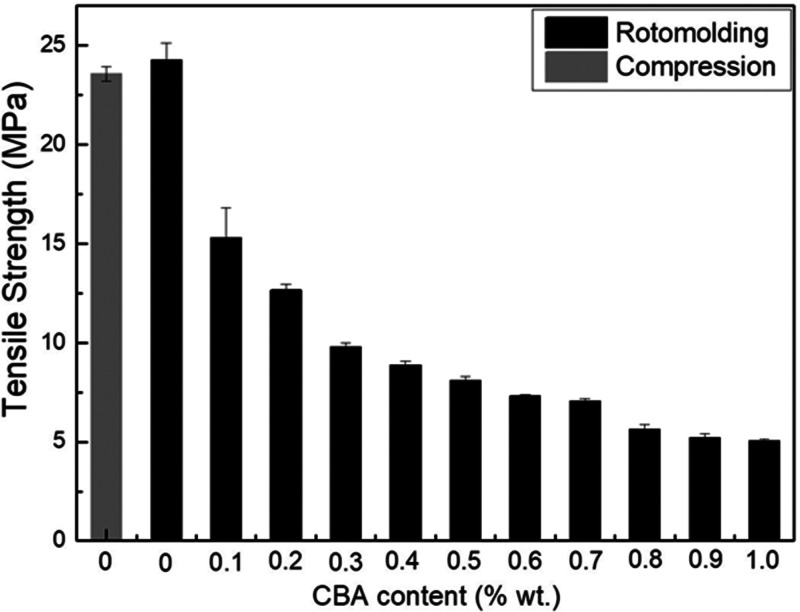
Tensile strength of rHDPE foams as a function of CBA content.

**Figure 11.**
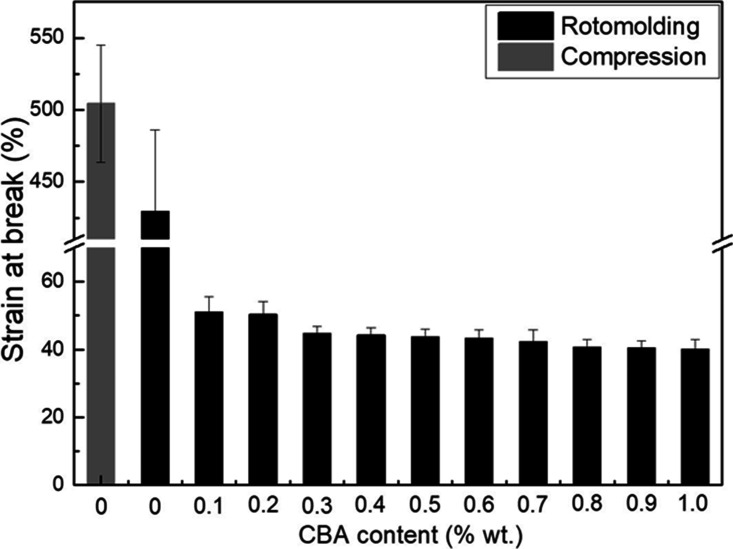
Tensile strain at break of rHDPE foams as a function of CBA content.

Figures 9 [Fig fig10-0021955X211013793]to 11 present the tensile properties of all the rHDPE samples. For the tensile modulus (Figure 9) and tensile strength (Figure 10), there are no statistically significant differences between the compression molded (302 ± 16 MPa) and rotomolded (273 ± 20 MPa) samples as for the flexural modulus (Figure 8), while the values for tensile strength are 23.6 ± 0.4 MPa and 24.3 ± 0.9 MPa, respectively. However, the elongation at break (Figure 11) of the compression molded rHDPE (504 ± 41%) is slightly better than that of rotomolded parts (429 ± 57%), which may be related to high pressure involved in compression molding leading to a better compaction (closer packing) reducing the number of microvoids in the samples.^39^ This result indicates that the differences between both processing methods (compression molding vs. rotomolding) are mainly important at higher deformation (elongation at break) compared to lower deformation (elastic modulus and maximum stress).

For the rotomolded foams, the tensile modulus (Figure 9) presents the same trend as for the flexural modulus (Figure 8). For example, the tensile modulus decreased by 68% (from 273 to 88 MPa) with increasing CBA content from 0 to 1% wt. Figure 10 reveals that the tensile strength also decreases with increasing CBA content, due to the same reasons as for the flexural modulus. For example, the tensile strength of the unfoamed matrix (24.3 MPa) decreased to 5.1 MPa (70% lower) at 1% wt. CBA. Finally, Figure 11 presents the results for the elongation at break. Again, the values decrease with increasing CBA content and are all well below 100% (40 to 50%), but do not change much. All these findings are consistent with other studies reporting decreasing mechanical moduli, strengths and deformations at break with increasing foaming level.^40^

### Impact strength

**Figure 12.**
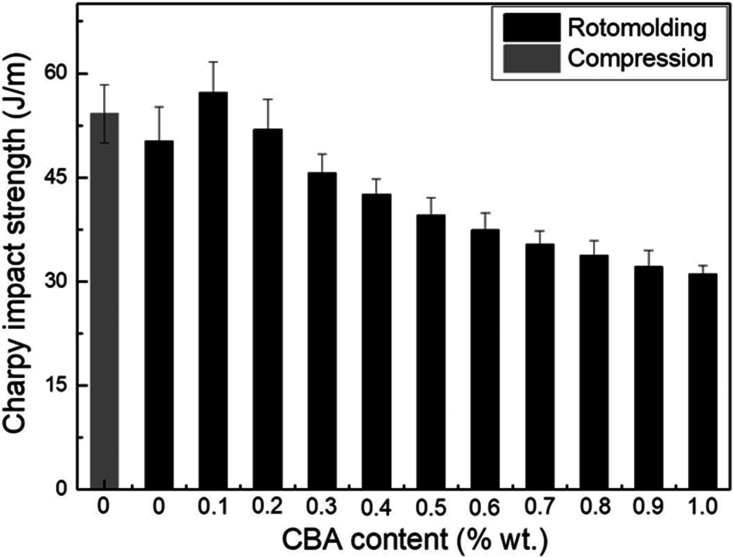
Impact strength of rHDPE foams as a function of CBA content.

Impact strength results are shown in Figure 12. Compared with the neat rHDPE (50.2 ± 4.9 J/m) in rotomolding, the value slightly increases in compression molding (54.2 ± 4.2 J/m) because of a more compact structure. This indicates again that the main difference between both processes is important at higher deformation rate (impact).

Compared with the unfoamed matrix, the impact strength slightly increases at 0.1% wt. (57.2 J/m) and 0.2% (52.0 J/m) CBA. This improvement may result from a finer cellular structure produced at lower CBA content and each (closed) cell acting as energy absorbers leading to higher impact strength.^41^ However, as the CBA content further increases (0.3 to 1% wt.), the cell density decreases and cell coalescence occurs (Figure 5 and Table 1). In this case, there is more defects in the samples leading to lower impact strength down to 31.1 J/m at 1.0% wt. CBA. It has been reported that larger cells are acting as stress concentration points and easier crack propagation occurs with decreasing cell wall thickness (larger cell sizes),^25^ which confirms our results.

## Final analysis

**Figure 13.**
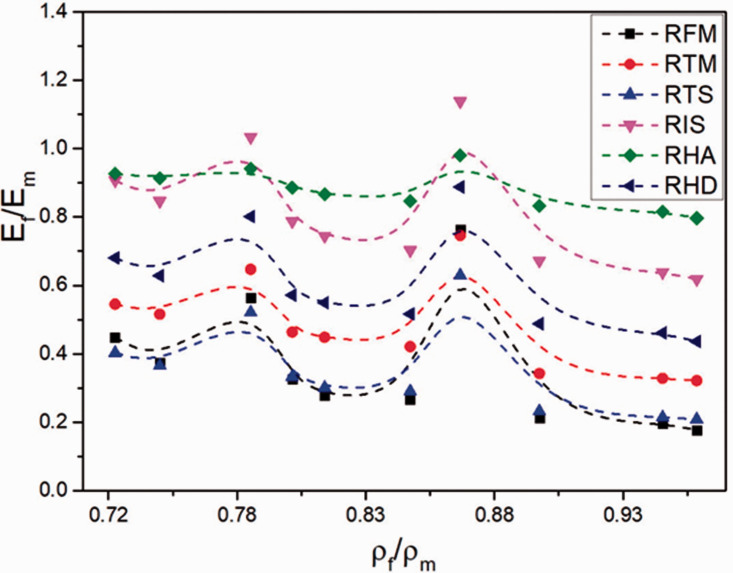
Plots of relative mechanical properties (E_f_: mechanical property of the foam divided by E_m_: mechanical property of the polymer matrix) as a function of the relative density (ρ_f_: density of the foam divided by ρ_m_: density of the polymer matrix). RFM: relative flexural modulus, RTM: relative tensile modulus, RTS: relative tensile strength, RIS: relative impact strength, RHA: relative Shore A hardness and RHD: relative Shore D hardness.

To complete our analysis, the relative mechanical properties (property of the foam divided by the property of the matrix) are plotted in Figure 13 as a function of the relative density (density of the foam divided by the density of the matrix) for the rotomolded rHDPE foams. It can be seen that no clear trend can be obtained in our case due to the complex interactions between all the parameters involved. This indicates that more parameters (cell size, cell density, open cell content, etc.) must be included to get a clear picture of these trends. Nevertheless, these plots are helpful to optimize a specific system (polymer, blowing agent, processing methods and conditions, etc.) depending on the final application of the foam. As always, a balance between maximum properties with minimum density must be achieved. Based on the results of Figure 13, it seems that the sample with a relative density of 0.862 g/cm^3^ (0.1% wt. CBA) gives the best results.

## Conclusions

In this study, post-consumer recycled high density polyethylene (rHDPE) foams were successfully produced via rotational molding using an initial dry-blend of a chemical blowing agent (CBA) in a powder form with pulverized rHDPE. Then, the effect of CBA content (0–1% wt.) was evaluated. Furthermore, according to the comparison of thermal and mechanical properties between compression-molded rHDPE parts and rotomolded rHDPE parts, the results showed that good processing conditions were used in rotational molding as the properties at low deformation and/rate of deformation were similar.

As for the rotomolded foam samples produced, a complete set of characterization including morphological, thermal and mechanical properties was performed. According to the results obtained, several conclusions can be made.

Firstly, based on DSC results, the polymer was completely melted before the CBA started decomposing and the oven temperature selected (270 °C) was able to produce good parts over the range of conditions tested after preliminary optimization.

Secondly, the morphological analysis indicated that the average cell diameter of the foamed rHDPE increased with increasing CBA content, while cell density initially increased and then decreased due to possible cell coalescence and gas loss. Moreover, the density firstly decreased, and then increased according to the cell density trend. As expected, due to the soft nature of the gas cells, the hardness decreased with CBA addition.

Thirdly, the thermal insulation properties of rHDPE foams were improved with increasing CBA content. The lowest thermal conductivity was 0.124 W/m^.^K at 1% wt. CBA, which is quite low for this relatively high density foam (0.7 g/cm^3^).

Finally, increasing the CBA content not only decreased both the tensile and flexural moduli, but also decreased the tensile strength and strain at break. For the impact strength, the values initially increased due to a fine cellular structure acting as energy absorbers, before decreasing due to larger cells acting as stress concentrators.

Nevertheless, the results obtained clearly indicates that more work is needed to optimize the processing of polymer foams based on recycled resins, especially to completely understand the relations between all the parameters involved on the final structure and properties.
